# A first near real-time seismology-based landquake monitoring system

**DOI:** 10.1038/srep43510

**Published:** 2017-03-02

**Authors:** Wei-An Chao, Yih-Min Wu, Li Zhao, Hongey Chen, Yue-Gau Chen, Jui-Ming Chang, Che-Min Lin

**Affiliations:** 1 Department of Civil Engineering, National Chiao Tung University, Hsinchu 300, Taiwan; 2 Department of Geosciences, National Taiwan University, Taipei 10617, Taiwan; 3 National Center for Research on Earthquake Engineering, National Applied Research Laboratories, Taipei 10668, Taiwan; 4 Institute of Earth Sciences, Academia Sinica, Nankang, Taipei 11529, Taiwan; 5 National Science and Technology Center for Disaster Reduction, Sindian, Taipei, 23143, Taiwan

## Abstract

Hazards from gravity-driven instabilities on hillslope (termed ‘landquake’ in this study) are an important problem facing us today. Rapid detection of landquake events is crucial for hazard mitigation and emergency response. Based on the real-time broadband data in Taiwan, we have developed a near real-time landquake monitoring system, which is a fully automatic process based on waveform inversion that yields source information (e.g., location and mechanism) and identifies the landquake source by examining waveform fitness for different types of source mechanisms. This system has been successfully tested offline using seismic records during the passage of the 2009 Typhoon Morakot in Taiwan and has been in online operation during the typhoon season in 2015. In practice, certain levels of station coverage (station gap < 180°), signal-to-noise ratio (SNR ≥ 5.0), and a threshold of event size (volume >10^6^ m^3^ and area > 0.20 km^2^) are required to ensure good performance (fitness > 0.6 for successful source identification) of the system, which can be readily implemented in other places in the world with real-time seismic networks and high landquake activities.

Landquakes such as rockfalls, landslides and rock avalanches are one of the most deadly kinds of natural disasters. In active mountain belts, such gravity-driven events dominate erosion dynamics that are closely linked to the occurrence of extreme rainfall and/or high seismicity^1^,^2^. The catastrophic Shiaolin landquake event in August 2009, which resulted in 465 deaths, occurred in the mountain area of southern Taiwan during intense and prolonged rainfall coinciding with the passage of Typhoon Morakot. The bursting of the short-lived (~104 minutes) Shiaolin landquake dam caused further serious flooding damage^3^. Rapid detection is therefore crucial for landquake hazard mitigation and emergency response. However, conventional techniques, such as optical remote sensing, are unable to provide any landquake information during a typhoon period with extreme weather conditions.

In general, seismic records can be used to investigate a wide variety of sources, such as earthquakes^4^,^5^, icequakes^6^, nuclear explosions^7^,^8^ and mine blasts^9^,^10^. Recent studies have demonstrated that seismic monitoring is also an effective technique to detect landquake events^11^,^12^,^13^,^14^,^15^,^16^ and capture the ground vibrations induced by river bedload transport^17^,^18^,^19^. For real-time monitoring purpose, an automatic scheme for the detection and identification of the landquake sources using seismic signals is needed. However, most landquake detection procedures proposed in previous studies are still not fully automatic. For example, time-frequency analyses of seismic records in previous studies have shown that landquake spectrograms exhibit a nearly triangular-shaped energy concentration in the frequency range of 1–3 Hz (refs 12, 20 and 21). However, due to the difficulty in quantifying the time-dependent spectral characteristics of signals influenced by source dynamics (e.g., higher frequency arriving later but decaying earlier), identification schemes based on pattern recognition of spectrograms for different landquake source types may not be easily implemented for real-time operation. Previous studies^8^,^9^,^10^ have also adopted systematic comparison of linear (Fisher discriminant analysis) and non-linear (random forests, support vector machine and naive Bayes classification) classifiers based on statistical machine learning for the identification of seismic events and blasts. To automatically detect rockslide events, a recent work^21^ proposed a classification approach using the hidden Markov models (HMMs), which provides a powerful tool to describe highly variable time series, allowing for a general description of signal classes. Most of the aforementioned classifiers that are based on a data-driven stochastic model perform well only when the characteristics used contain different information for different classes. In this study, we develop a near real-time landquake monitoring system (NRLMS) in which we adopt a general source inversion (GSI) technique^22^ (see Methods) with deterministic full waveform modeling as the identification module. GSI can effectively identify landquake sources by observing the improvement in waveform fitness with the consideration of different types of source mechanisms, including full, deviatoric and isotropic moment tensors (fMT, dMT and iMT, respectively), and single-force (SF) mechanism. In the future, the aforementioned statistical classifiers can be readily incorporated into the NRLMS as additional source classification modules.

Our aim here is to introduce the NRLMS, a system for fully automatic detection and identification of landquake events, determination of source locations and mechanisms, and the online dissemination of the results. Using records from existing real-time broadband seismic networks, the NRLMS can automatically detect and identify landquake sources by combining the grid-based single-force (gSF, see Methods) inversion and the GSI procedures. Following the detection and identification modules, the NRLMS also consists of modules for source location and dynamics estimation, as shown in the flowchart in Fig. 1. In summary, the NRLMS can automatically detect and identify landquake events, and provide simultaneously the event locations, occurrence times and the force mechanisms (magnitude, dip and direction of the sliding block-mass).

## Results

### Grid-based single-force inversion and general source inversion

**Figure 1:**
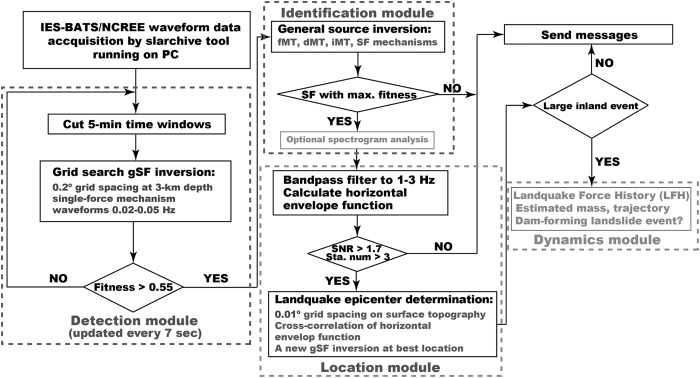
Flowchart of near real-time landquake monitoring system (NRLMS). The system consists of modules for landquake detection, identification, location and dynamics estimation. Optional procedures (spectrogram analysis and dynamics module) marked by the light grey colors are not implemented in the automatic system.

**Figure 2:**
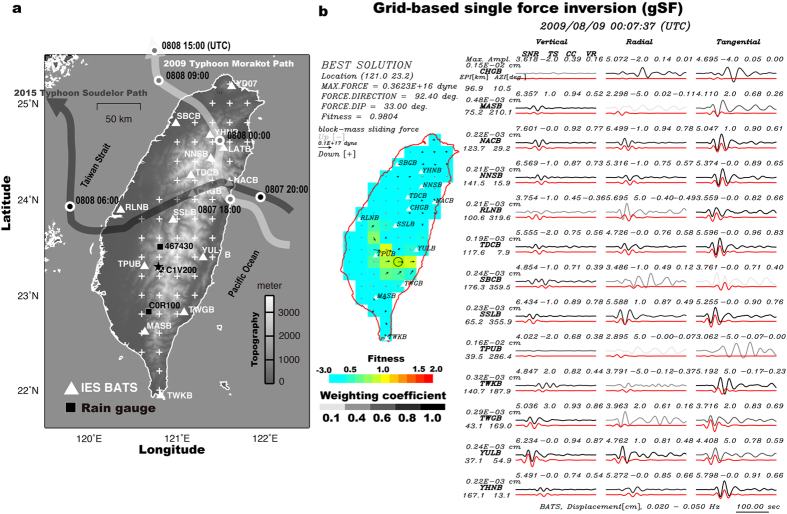
Station distributions and grid-based single force (gSF) inversion. (**a**) Distributions of broadband seismic stations (triangles), points for grid search (crosses) in gSF inversion, and rain gauge stations (squares). The two thick lines with arrows depict the paths of the 2009 Typhoon Morakot and the 2015 Typhoon Soudelor. Filled and open stars show the locations determined by LED procedure using IES-BATS stations only and using both IES-BATS and NCREE stations, respectively. (**b**) Example of gSF inversion for a landquake event (Event No. 17 in Table S2) detected by the NRLMS offline test. Left panel summarizes the results of source location and the maximum magnitude, direction and dip of the sliding force. Color scale on the map represents the waveform fitness, with the black circle indicating location of the largest fitness value (0.9804). Right panel displays records (grey curves) and synthetic seismograms (red curves) calculated for the best single-force solution. Different grey levels indicate different weighting factors based on signal-to-noise ratios (SNR) of individual records (Table S1). All waveforms are bandpass filtered to 0.02–0.05 Hz. The maximum amplitude, station name, epicentral distance and station azimuth are given at the start of each row. The SNR value, time shift (TS), normalized cross-correlation coefficient (CC) and variance reduction (VR) are given at the top of each trace. Maps are created using GMT (Generic Mapping Tools, http://gmt.soest.hawaii.edu/; ref. 23) software.

In detecting landquake events, we first assume a single-force (SF) mechanism and conduct a grid-based single-force (gSF) inversion (see Methods) using long-period (LP; 20–50 s) waveforms by searching through all grid points (crosses in Fig. 2a) to find the best-fit solution. Figure 2b shows the example of a gSF solution (Event No. 17 in Table S2) with a waveform fitness value of 0.9804, indicating a block-mass sliding force with an azimuth of 92.4° (clockwise from north), a force magnitude of 3.623 × 10^15^ dyne, and a preliminary location at (121.0°E, 23.2°N). Following this positive result by the detection module, the NRLMS triggers the identification module (Fig. 1) and applies the GSI (see Methods) to identify the landquake event. Figure S1 shows the fits between records and synthetic seismograms calculated for different source mechanisms including a full moment tensor (fMT; fitness: 0.6968) as well as deviatoric and isotropic moment tensors (fitness values of 0.6799 and 0.7268 for dMT and iMT, respectively). Indeed, SF mechanism yields the best fitness (0.9804) for this landquake event, which demonstrates that in this case successful identifications of landquake sources in our NRLMS can be achieved by simply comparing the waveform fitness values in the gSF inversion in the detection module and the GSI in the identification module.

### Offline Test

**Figure 3:**
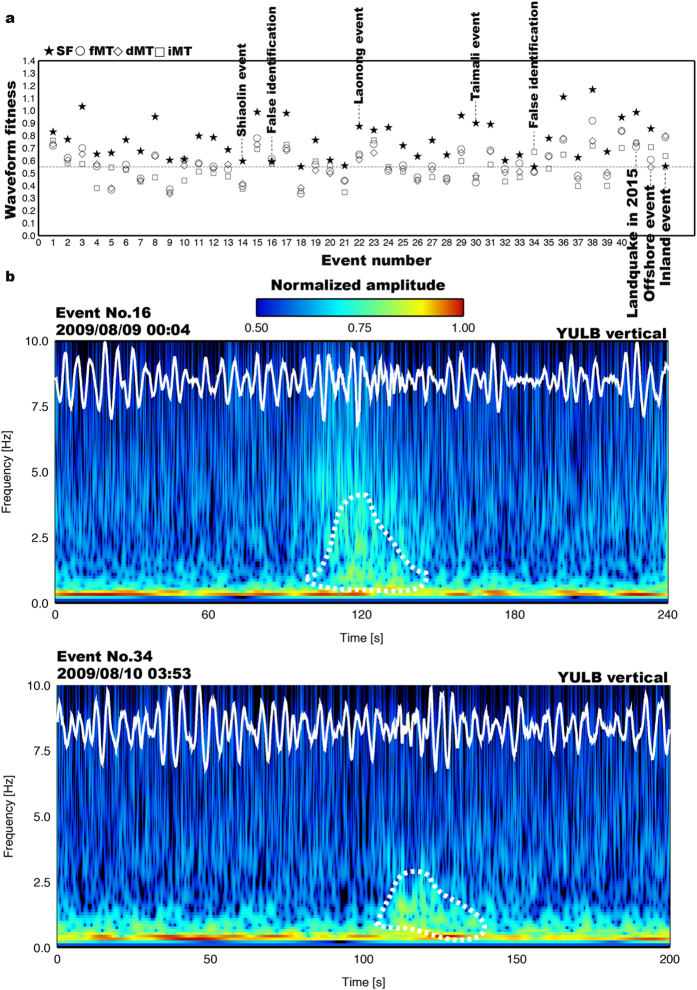
General source inversion (GSI) and spectrograms. (**a**) Waveform fitness from different source mechanisms including single force (SF, solid star), full (fMT, open circle), deviatoric (dMT, open diamond) and isotropic (iMT, open square) moment tensors. The horizontal dashed line indicates the threshold value of 0.55 in the detection module. (**b**) Spectrograms for Event Nos 16 and 34 in Table S2. White traces are the original vertical-component velocity seismograms. On the time-frequency plane the highest concentrations of energy arriving from the event are marked by the white dashed lines. The color scale is such that the maximum normalized amplitude is depicted in red while black indicates normalized amplitudes less than 0.5.

An offline test of the NRLMS was initially carried out using seismograms recorded during the passage of the 2009 Typhoon Morakot. Using a threshold fitness value of 0.55, the system automatically detected a total of 40 landquake events with maximum force magnitudes (*F*_max_) between 0.358 × 10^15^ dyne and 18.570 × 10^15^ dyne and a waveform fitness range of 0.5509–1.1679 (Table S2). In the identification module, most events were successfully identified as landquake sources by the GSI. For Event Nos 16 and 34, which were identified incorrectly as a tectonic event and an isotropic source, respectively (Fig. 3a), only a few stations had SNR ≥ 5.0 to perform the GSI. This suggests that waveform fitness from GSI is not sensitive to different source mechanisms when the gSF result from the detection module has a small fitness value (0.55–0.60). In the case of these two failed events, the optional spectrogram analysis in the identification module is an alternative tool to correctly classify them as landquake events by observing the triangular-shaped spectrograms (Fig. 3b,c). In practice, manual confirmation using spectrogram characteristics takes only a few seconds and can easily be included as a part of the identification module in the NRLMS (Identification Module in Fig. 1). Further investigation is needed to develop other automatic identification schemes (e.g., identifier using the HMMs) based on time-frequency characteristics. It is expected that most events with false identifications can be easily removed by increasing the threshold fitness value (threshold 0.55 used in the current NRLMS). However, the capability of NRLMS in detecting smaller landquake events will be reduced. For example, when adopting a threshold value of 0.60, a catastrophic Shiaolin event with a fitness of 0.5968 (Event No. 14 in Table S2) would not be detected. Since the main purpose of the NRLMS is to provide source parameters for catastrophic/large landquake events, we adopt a threshold value of 0.55 in the NRLMS.

**Figure 4:**
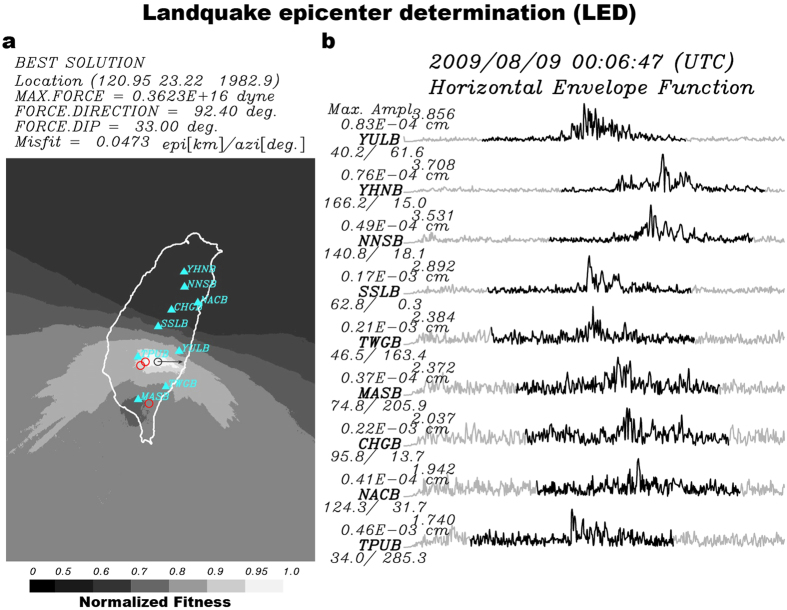
Landquake epicenter determination (LED). (**a**) Location determined from the LED method. The grey scale shows the normalized fitness value. Cyan triangles indicate seismic stations used in the LED location process. Black circle indicates location from LED with final solution of SF inversion, while red circles are locations of relatively larger events (Event Nos 14, 22 and 30) from field observation^12^. (**b**) Filtered horizontal envelope functions of landquake event. Black traces (100-s long time window) are used in the LED location method. The station name, epicentral distance, and station azimuth are given at the start of each trace. Misfit is calculated from the weighted sum of the cross-correlation amplitude differences proposed by Chen *et al*.^12^. Map is created using GMT (Generic Mapping Tools, http://gmt.soest.hawaii.edu/; ref. 23) software.

Among the 40 detected events listed in Table S2, 19 events were relocated by the landquake epicenter determination (LED, see Methods), with maximum source-to-receiver distances between 137.4 km and 232.6 km and the number of stations used between 3 and 10. The LED adopts a cross-correlation approach, which maximizes the coherence of the horizontal envelope function of seismic records, to determining the location more accurately. Figure 4 shows the LED result for the event in Fig. 2b (Event No. 17 in Table S2). After the LED relocation, a new SF inversion is conducted to update the force mechanism. In the offline test of NRLMS, only 19 events that are recorded by at least three stations with SNR values exceeding 1.7 in the high-frequency envelope functions could be located by the LED. Events without LED results may not have generated enough energy at the 1–3 Hz frequency range, which is generally caused by the impact of large blocks on a topographic barrier^12^,^24^.

**Figure 5:**
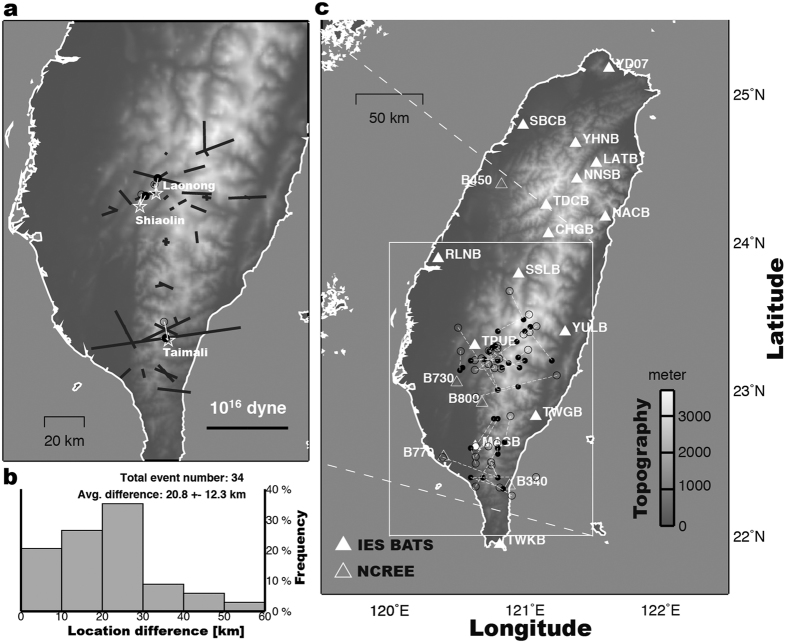
Distributions of stations and landquake events with force directions. (**a**) Directions of sliding force axes. (**b**) Histogram of the location difference between our result and *Lin et al*.^15^. (**c**) Distributions of IES-BATS (solid triangles) and NCREE (open triangles) seismic stations and landquake events determined by offline NRLMS (black dots) and Lin *et al*.^15^ (open circles). Open stars indicate the locations of three relatively large events (Event Nos 14, 22 and 30) from field observation^12^. Maps are created using GMT (Generic Mapping Tools, http://gmt.soest.hawaii.edu/; ref. 23) software.

**Figure 6:**
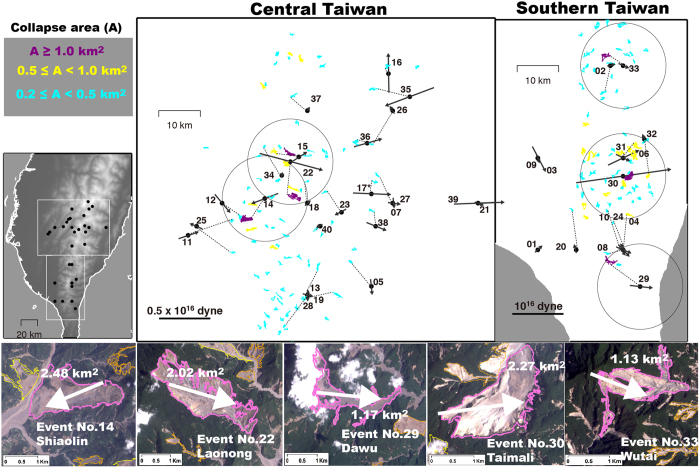
Event locations and satellite-image mapping results. Upper panel: Distribution of landquake events with force directions. Areas marked by purple, cyan and yellow colors indicate landquakes with collapse area ranges of A_C_ ≥ 1.00 km^2^, 0.50 ≤ A_C_ < 1.00 km^2^ and 0.20 ≤ A_C_ < 0.50 km^2^, respectively. Big circles indicate the searching radius of 10 km. Lower panel: Maps of the FORMOSA-2 satellite images with 2-m ground resolution derived by the Center for Space and Remote Sensing Research (CSRSR; http://www1.csrsr.ncu.edu.tw/Ver13_J30/). Regions marked in purple show the collapse areas of the Shiaolin, Laonong, Dawu, Taimali and Wutai events, as mapped by the Central Geological Survey of Taiwan (CGS). Arrows depict the sliding force directions of landquake events determined in this study. Maps are created using GMT (Generic Mapping Tools, http://gmt.soest.hawaii.edu/; ref. 23) software.

Locations of some of the detected events have been published previously with identification from satellite-image mapping^12^,^22^. The NRLMS can further provide the force magnitudes as well as the directions of the block-mass sliding, as shown in Fig. 5a. The available satellite-image dataset^12^, which consists of areas of collapse events mapped by the Central Geological Survey (CGS) of Taiwan before and after Typhoon Morakot, reveals 156 events with collapse areas (A_C_) larger than 0.20 km^2^ in the mountain area of southern Taiwan (Fig. 6). Based on the collapse events from the satellite-image mapping, the force mechanisms (direction and maximum magnitude of sliding force) and the 10-km searching radius around landquake centroid location, we correlate 34 out of the initial 40 detected landquake events with the mapped collapse areas. The landquakes detected here can be roughly associated with A_C_ values between 0.20 and 2.48 km^2^, with distances between the satellite-image mapped collapse areas and NRLMS locations ranging 0.6–9.2 km. Six events (Event Nos 1, 3, 7, 9, 21, and 39 in Table S2) have less than three stations with sufficient SNR values to perform the LED relocation procedure. These events may have larger location errors and thus could not be associated with mapped areas. For relatively large events with A_C_ values above 1.00 km^2^ (Event Nos 14, 22, 29, 30 and 33 in Table S2), our results of the force directions are generally consistent with field observations and satellite-image mapping results^12^ (Fig. 6). Most of the relatively large events mentioned here exhibit sliding axes in a west-east trend, which is generally perpendicular to the strike of the mountain belt.

### Online Real-time Operation

**Figure 7:**
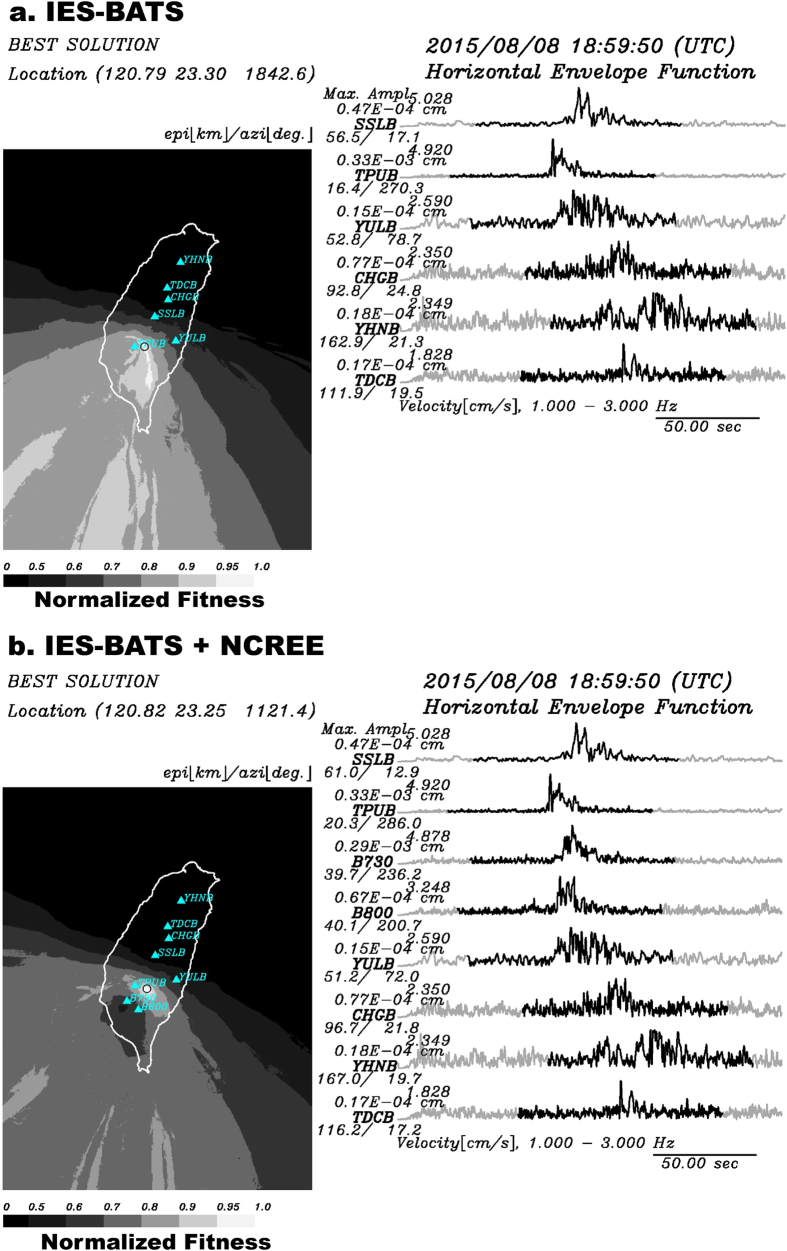
An online near real-time LED results. Locations determined by the LED using (**a**) IES-BATS only and (**b**) both IES-BATS and NCREE broadband seismic stations. The grey scale shows the normalized fitness value. Cyan triangles indicate seismic stations used in the LED location process. Black circle indicates the final location. Black traces indicate the filtered horizontal envelope functions of the landquake event. Black traces (100-s long time windows) are used in the LED location method. The station name, epicentral distance, and station azimuth are given at the start of each waveform. Maps are created using GMT (Generic Mapping Tools, http://gmt.soest.hawaii.edu/; ref. 23) software.

After the successful offline test, our NRLMS was put online in real-time operation during the passage of Typhoon Soudelor (Fig. 2a) on August 7–10, 2015, which dropped ~400 mm of rainfall on August 8 at the rain gauge station C1V200 operated by the Central Weather Bureau (CWB) of Taiwan (ref. 25, Figure S2). Typhoon Soudelor has a smaller magnitude relative to the 2009 Typhoon Morakot. Only one landquake event was successfully detected by the gSF inversion in NRLMS with a *F*_max_ value of 0.590 × 10^15^ dyne and a fitness value of 0.9859 (Figure S3). Using records from the Broadband Array in Taiwan for Seismology (BATS) stations maintained by the Institute of Earth Sciences (IES) of Academia Sinica (dubbed IES-BATS), the LED relocation module puts the event at (120.79°E, 23.30°N) (Fig. 7a; black star in Fig. 2a). After the LED relocation, the *F*_max_ value is updated as 0.481 × 10^15^ dyne by a new SF inversion. Notably, a change of ~22 km between the relocated source location from LED and gSF-determined location (Figure S3) leads to ~18% decrease in *F*_max_. However, the force direction of the block-mass seems not sensitive to the location difference, with only a 4.90° change. Based on the FORMOSAT-2 satellite images from the Center for Space and Remote Sensing Research (CSRSR) with a resolution of 2 m before and after Typhoon Soudelor, this event can be confirmed by the geomorphic change at (120.7769°E, 23.2131°N) with a collapse area of 0.21 km^2^ (Figure S4) near the Laonong event (Event No. 22 in Table S2 and Fig. 5a). The location difference between the satellite-image mapped collapse area and LED result is ~9.7 km. The force of the sliding mass points in the southwest direction (228.74° clockwise from north), consistent with the geomorphic change seen from satellite images (arrow in Figure S4). In addition, a clear triangular-shaped pattern can be seen in the spectrograms (Figure S5). We have also checked the CWB earthquake catalog, and this landquake event was not listed there.

During Typhoon Soudelor, an offshore earthquake was reported by the Central Weather Bureau (*M*_L_ = 4.2; http://www.cwb.gov.tw/V7/earthquake/rtd_eq.htm, last accessed August 2016). The NRLMS also detected this event and incorrectly classified it as an inland landquake event (annotated offshore event in Fig. 3a). False identification was mainly caused by the location error since gSF procedure of the NRLMS was designed only for monitoring inland landquake activity (Figure S6a). After conducting the LED procedure, this event was successfully relocated to the offshore region (Figure S6b). Indeed, the NRLMS did not report this offshore event (Fig. 1). A time-frequency analysis shows that this event has a typical earthquake spectrogram, which has seismic energy of a wide frequency range appearing after the onset of the first arrival and decaying exponentially (Figure S6c). For inland earthquakes in Taiwan, the NRLMS can yield successful identification by using the GSI procedure (annotated inland earthquake in Fig. 3a). Clearly, the current system can be used effectively not only to detect landquake sources but also monitor shallow earthquakes (Figure S7).

## Discussion

**Figure 8:**
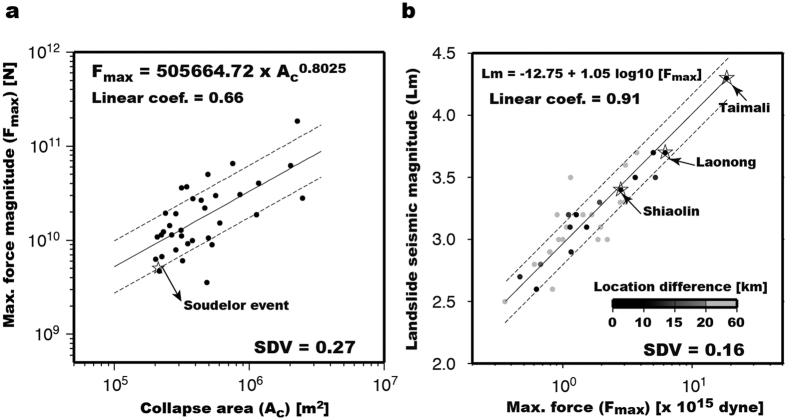
Regression scaling relations. (**a**) Maximum force (*F*_max_) versus collapse area (A_C_) and (**b**) Landslide seismic magnitude (*L*m) versus *F*_max_. The black solid line is the regression line, and the two dashed lines show the one standard deviation (SDV). Grey scale for the symbols indicates location difference between this study and ref. 15.

In general, regional 1-D velocity models can be effective in modeling seismic waveforms of relatively long-period signals. The LP (20–50 s) signals used in gSF procedure of the NRLMS have wavelengths of a few hundred kilometers, thus the effect of lateral structural variations and attenuation can be discounted. For this reason, the size estimation (i.e., force magnitude) of landquake events should not be strongly influenced by the effect of 1-D velocity models. Assuming that the *F*_max_ values derived in this study correspond to the total energy release of landquake events, we would expect a logarithmic linear scaling between *F*_max_ and mapped collapse area (A_C_). To test our hypothesis, we conducted a linear regression analysis and the result showed a linear fit with a correlation coefficient of 0.66 with a standard deviation (SDV) of 0.28 (Fig. 8a). Result of the Typhoon Soudelor landquake event (open star in Fig. 8a) detected in the online operation of NRLMS roughly follows this relationship between A_C_ and *F*_max_. A recent study^15^ proposed a landslide magnitude (*L*m), which is defined based on the peak ground displacement and source-to-receiver distance. Since both the maximum force magnitude *F*_max_ obtained in our waveform inversion and the landslide magnitude *L*m are estimated from the LP seismic signals, we can expect that a larger *F*_max_ corresponds to a larger *L*m. Indeed, the comparison of these two magnitudes in Fig. 8b shows a high linear correlation coefficient of 0.91 with a SDV of 0.16. However, the *L*m estimation may be more prone to error for relatively small events with poor station coverage due to higher uncertainty in the source location determined from the arrival-time-based method. In contrast, the gSF waveform inversion in the NRLMS can achieve a reliable solution (waveform fitness > 0.60 with successful identification in GSI) using only a few stations (6 waveform traces from at least 3 stations; see Methods) with good SNR values (≥5.0). The average difference between the source locations from our gSF waveform inversion and the arrival-time-based method is 20.8 km with a SDV of 12.3 km (Fig. 5b,c). Figure 8b shows clearly that events with larger location differences (grey dots) between the two methods have greater discrepancies between the *F*_max_ and *L*m values. Generally, larger events can be recorded with better station coverage (station gap ≤180°, defined as the largest azimuthal gap between azimuthally adjacent stations) and sufficient SNR values, for which both arrival-time- and waveform-based location methods work well. As shown in Fig. 5a, for the relatively large-sized events (Nos 14, 22, and 30 in Table S2; associated A_C_ ≥ 2.00 km^2^ in Fig. 6), the location differences are less than ~8 km, and both locations (open and solid circles in Fig. 5a) are close to those inferred from satellite-image mapping and field observation^12^.

In the current system, relocation results from the LED module can also be strongly influenced by the distribution of seismic stations. For the landquake detected during Typhoon Soudelor (Fig. 7a), the IES-BATS used in the LED method for the same event has a large station gap (298°). To improve the station coverage, an additional 5 broadband stations (open triangles in Fig. 5c) maintained by the National Center for Research on Earthquake Engineering (NCREE) of Taiwan were included in the current system (see Methods). The well-distributed stations (with a minimum inter-station distance of 34 km) from both the IES-BATS and the NCREE networks provide sufficient coverage in distance (with a range of 39–167 km) and azimuth (station gap of about 136°), which ensures more accurate locations with lower uncertainties, as shown for the same landquake event in Fig. 7b. In Fig. 7, areas of higher fitness (≥0.95) are shown in lighter shades, indicating a higher probability of the source location. Indeed, this landquake event was relocated to (120.82°E, 23.25°N), ~5.9 km away from the mapped collapse area from satellite images, closer than the location using IES-BATS stations only.

**Figure 9:**
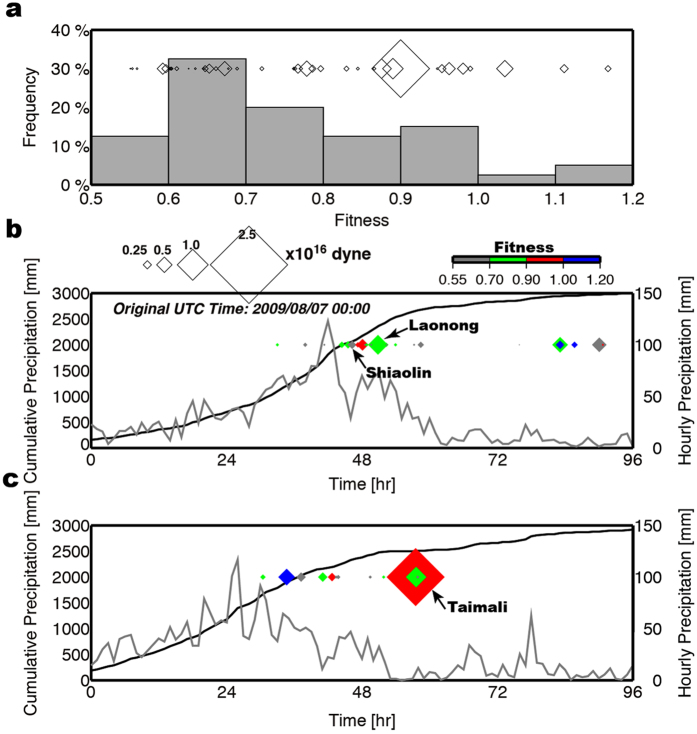
Relationships between precipitation, waveform fitness and landquake events. (**a**) Top panel is the histogram for the fitness values from waveform inversion. Size of diamonds represents the magnitude of landquake sliding force. Lower two panels show the time-series of precipitation rate (grey) and cumulative rainfall (black) at two rain gauge stations (**b**) 467530 (middle) and (**c**) C0R100 (bottom) during the 2009 Typhoon Morakot passage. See Fig. 2a for the locations of the rain gauge stations. Fitness values are indicated by the colors of the diamonds.

Naturally, the success of event detection depends on the background noise level as well as the size of landquakes. In particular, the NRLMS can only detect landquake sources that generate sufficiently strong LP seismic signals used in gSF modeling with the block model approximation. In order to evaluate the capability of the current seismic network in detecting landquakes, we examine the relationship between waveform fitness and event size. Figure 9a shows that the fitness values of waveform inversions range between 0.55 and 1.20, with information on the maximum force magnitude (*F*_max_). In general, seismograms from smaller events (*F*_max_ < 10^15^ dyne and *L*m < 3.0) may have relatively poor SNR values at long periods, and the SNR value usually also decreases with increasing source-to-station distance. Some of the events detected in this study have relatively small waveform fitness values due to several different factors. First, the signals produced by small events are not strong enough (SNR < 5.0) for LP waveform modeling. Second, a SF mechanism does not take finite-source effect into account. For example, the two largest events do not have the highest fitness values (Event Nos 22 Laonong and 30 Taimali with fitness values of 0.8752 and 0.9003, respectively), which implies that only a SF mechanism cannot fully model the source characteristics of relatively large events. Consideration of multiple-force mechanism is needed. To resolve this issue, we can adopt the landquake force history (LFH) inversion technique developed in two recent studies^13^,^22^ to investigate the dynamic source processes using multiple time-dependent forces (dynamics module in Fig. 1). Current NRLMS does not involve the LFH procedure in the real-time implementation. Details of the dynamics for three larger events with A_C_ >2.00 km^2^ (Event Nos 14, 22 and 30) can be found in Chao *et al*.^22^. After conducting the LFH inversion, the fitness values of three events were significantly improved (Table S1 in ref. 22). Third, landquake events are often triggered by heavy rainfall, and the higher ambient noise level during extreme weather conditions can also impede the capability of broadband seismic networks in detecting landquake sources. Moreover, for a rapid determination of the collapse mass (*m*), we can simply use an empirical linear relationship established by Chao *et al*.^22^ (*m* = 0.405*F*_max_) to estimate the block mass using the force magnitude obtained by the NRLMS. Based on the relationship between *m* and *F*_max_, the estimated collapse mass of the smallest event detected in this study is 0.145 × 10^10^ kg. Assuming an average density of 2500 kg/m^3^, the estimated collapse volume is 0.580 × 10^6^ m^3^, which may be considered as the lower limit of the detection capability by the current system. Thus, our NRLMS can only be used to detect large and fast landquake sources (volume > 10^6^ m^3^ and area >0.20 km^2^) that generate LP seismic signals with sufficient SNR values. In order to improve the ability of detection, especially for relatively small landquake events, deployment of more broadband seismic stations is needed.

Precision in the occurrence time enables us to explore the temporal link between the seismologically detected landquake events and rainfall data. In Fig. 9b,c and Figure S2, we compare the rainfall data during the 2009 Typhoon Morakot at two rain gauge stations 467430 and C0R100 in central and southern Taiwan, respectively, and at the rain gauge station C1V200 during the 2015 Typhoon Soudelor (Fig. 2a), with the landquake events detected nearby. The comparison shows that most events detected by our NRLMS occurred during the typhoon period with the most intense and prolonged rainfall. The largest one (Taimali event in Fig. 9c, or Event No. 30) occurred during the time period with a cumulative rainfall reaching 2500 mm. Only a few landquakes were detected in the mountain area of central Taiwan late on August 10 with obvious reduction in cumulative rainfall (Fig. 9b). A proposed mechanism leading to rapid mass-movement is the increase in the pore pressure in the sliding material leading to partial liquefaction. Consequently, Event Nos 14 and 22 (Shiaolin and Laonong, respectively) occurred a few hours after the peak rainfall and may be triggered by the extreme conditions with a precipitation rate of over 29 mm/hr and an accumulated rainfall of over 2050 mm (Fig. 9b).

In real-time monitoring, once the seismic waves reach a number of stations, it takes only ~7 s for the NRLMS to perform the waveform inversion and locate the source on a workstation (e.g. a MacBook Pro). The total system latency is mainly controlled by the length of the time window used in the waveform inversion. Starting in 2015 under the support of the Ministry of Science and Technology (MOST) of Taiwan, the NRLMS has been continuously monitoring landquake activity in Taiwan, which provides a complete landquake catalog for the comprehensive landtoring (landquake monitoring) laboratory at the National Taiwan University (NTU CoLLab, http://140.112.57.117/main.html). After receiving waveform data (5-min. long time window) from a number of stations and completing all the modules from waveform inversion to LED, the current NRLMS delivers a landquake report to users via e-mail automatically within ~6 min. after the occurrence of an event. The main goal for the NRLMS is to provide landquake source information in near real-time for rapid landquake hazard assessment and emergency response. Our proposed NRLMS can be readily implemented in other places with frequent landquake occurrence and high-density real-time broadband seismic networks, such as Japan and Italy.

## Methods

### Data

Real-time broadband waveforms used in this study come from 15 stations of the Broadband Array in Taiwan for Seismology (BATS, http://bats.earth.sinica.edu.tw/) maintained by the Institute of Earth Sciences (IES) of Academia Sinica, Taiwan. The IES-BATS stations are equipped with STS-1/STS-2/Tillium-240 seismometers and Q330HR dataloggers. The instrument responses can reach periods of 360 s, 120 s, and 240 s for STS-1, STS-2 and Trillium-240, respectively. 5 real-time seismic stations equipped with Guralp CMG-6TD intermediate band seismometer with a flat response in the 0.033–100 Hz frequency band and operated by the Taiwan National Center for Research on Earthquake Engineering (NCREE) are added to the IES-BATS stations in the NRLMS to enhance the station coverage. Both IES-BATS and NCREE stations have a sampling rate of 100 Hz. For continuous data flow processing, the NRLMS acquires the raw data from the IES-BATS and NCREE database servers, which broadcast the real-time broadband data in SEED format, via ‘slachive’ developed by the Incorporated Research Institutions for Seismology (IRIS). The NRLMS performs a series of data-processing operations, including converting the data format from SEED to SAC (http://ds.iris.edu/files/sac-manual/), integrating from ground velocity to displacement, rotating the horizontal records to radial and transverse components, cutting the data into time windows of 5-min. length, applying a fourth-order minimum-phase Butterworth band-pass filter, and resampling the waveforms to 10 Hz.

### Grid-based single-force (gSF) inversion

The single-force inversion algorithm is proposed in Chao *et al*.^22^, which uses full-waveforms in a grid search over the monitoring area for the best-fitting location and single-force (SF) mechanism. A 2-D grid with an interval of 0.2° in both latitude and longitude is created in the mountain area of the Taiwan Island. Different weightings are assigned in the inversion based on the signal-to-noise ratios (SNR) of the filtered waveforms, as shown in Table S1. The SNR is computed from the ratio between the absolute peak amplitude and the whole-trace average of the absolute amplitude. The synthetics are obtained using Green’s functions computed by the propagator matrix approach^26^ for a 1-D average velocity model^27^. Each grid-point to station pair has nine Green’s function elements corresponding to six elementary moment tensors and three orthogonal (north, east and vertical) forces, which are contained in a Green’s function database and stored on the hard disk for rapid synthetics calculation in the inversion. The 1-D velocity model does not account for surface topography, which may be up to 3 km in the mountain area of Taiwan. In general, regionally averaged 1-D models and a grid spacing of 0.2° are sufficient in modeling relatively long-period waveforms. In Taiwan, Green’s functions based on a 1-D velocity model^27^ can work sufficiently well for a real-time moment tensor determination system^28^ (RMT) by using the long-period waveform data between 10 and 50 s for automatically monitoring earthquake activities. Thus, we fix the depth of all grid points at 3 km, which does not affect the inversion results significantly since all waveforms in the inversion are band-pass filtered to periods between 20 s and 50 s. Using the different depths (0 and 3 km) for grid points, there is only ~5% difference in the resulting maximum force magnitude (*F*_max_). In future works considering the effects of lateral structural heterogeneity and surface topography, we can use Green’s functions numerically computed in 3-D tomography models with realistic surface topography^29^. If the number of waveforms used is greater than 6 and the waveforms are from at least 3 stations, the NRLMS proceeds to the gSF inversion in the detection module (Fig. 1). Finally, the direction, dip and magnitude of three orthogonal (north, east and vertical) forces are solved by minimizing the misfit between observed and synthetic seismograms. Currently the system can complete one grid search in gSF waveform fitting throughout the monitoring area every 7 s in real-time. If no event is detected, the system updates the real-time data and conducts a new search (Fig. 1). In the detection module, we have experimented with different threshold values for waveform fitness and found that a value of 0.55 yields a high successful classification rate (95%) for the event with maximum force magnitude larger than ~0.35 × 10^15^ dyne. The details are discussed in the Offline Test section.

### General source inversion

After a preliminary location and single-force (SF) mechanism is determined, the NRLMS automatically identifies the landquake source by conducting a more flexible approach (general source inversion^22^, GSI), which models the source mechanism as a full moment tensor (fMT, combining effects of volume change, tensile crack and faulting), deviatoric MT (dMT), isotropic MT (iMT, explosion source), and SF (block mass sliding), to identify landquake events by examining waveform fitness values. A time shift of up to ±5 s is allowed independently for each component to achieve the maximum normalized cross-correlation coefficient (CC) value. The waveform fitness between synthetic and observed seismograms is defined by both the variance reduction (VR) and CC values. The best fit coefficients corresponding to moment tensors and SF mechanism are individually determined in a least-squares scheme (equation (1) in ref. 22). The synthetics and observed seismograms are filtered in the same way as the gSF inversion. Only vertical and radial components were used in the waveform modeling for the iMT source.

### Landquake epicenter determination (LED)

To determine the source location more accurately than the preliminary location from the gSF inversion, the NRLMS has a location module that conducts the landquake epicenter determination (LED) procedure (ref. 12) to relocate the landquake source by a grid-search inversion. The location module in the NRLMS first calculates the root-mean-square (RMS) amplitudes of the high-frequency (1–3 Hz) horizontal-component waveforms to create horizontal envelope functions by considering a specific time window (±50 s from the peak envelope amplitude, black trace in Fig. 4b). Then a cross-correlation-based location method^12^ is used to find the best location with the maximum coherency of envelope functions among stations. At least three envelope functions with SNR ratios larger than 1.7 are considered in the LED procedure. Here the SNR is calculated from the ratio between short-term (±5 s from the peak envelope amplitude) average and long-term average (±50 s from the peak envelope amplitude). In a previous study^12^, only waveforms with SNR ratio (between peak envelope amplitude and whole-term average) larger than 2.5 are used in the LED procedure. Here, we adopted a different SNR definition and found by experimentation that a threshold SNR value of 1.7 yields location result similar to the previous work^12^ for the Shiaolin event. In the grid-search inversion, the grid points are located on the surface topography in the region of longitude from 118.5°E to 123.5°E and latitude from 20.0°N to 26.5°N, with a grid spacing of 0.01°. We also define a weighting factor in the optimization process based on the values of cross-correlation coefficient (CC) between envelopes of each station pair, as shown in Table S3. Misfit is calculated from the weighted sum of the cross-correlation amplitude differences (equation (3) in ref. 12). The normalized fitness value shown in Fig. 4 is defined in equation (4) of Chao *et al*.^12^.

## Additional Information

**How to cite this article:** Chao, W.-A. *et al*. A first near real-time seismology-based landquake monitoring system. *Sci. Rep.* **7**, 43510; doi: 10.1038/srep43510 (2017).

**Publisher's note:** Springer Nature remains neutral with regard to jurisdictional claims in published maps and institutional affiliations.

## Supplementary Material

Supplementary Information
