# Supplementary material for: A first near real-time seismology-based landquake monitoring system
Source: Sci Rep. 2017 Mar 2;7:43510. doi: 10.1038/srep43510 (PMC5333350; doi:10.1038/srep43510)
Supplement: Supplementary Information [file srep43510-s1.pdf]

Supplementary Information

A first near real-time seismology-based landquake monitoring system

Wei-An Chao[1*], Yih-Min Wu[1,2], Li Zhao[3], Hongey Chen[1,4], Yue-Gau Chen[1], Jui-Ming Chang[1] and Che-Min Lin[2]

1. Department of Geosciences, National Taiwan University, Taipei 10617, Taiwan
2. National Center for Research on Earthquake Engineering, National Applied Research Laboratories, Taipei 10668, Taiwan
3. Institute of Earth Sciences, Academia Sinica, Nankang, Taipei 11529, Taiwan
4. National Science and Technology Center for Disaster Reduction, Sindian, Taipei, 23143, Taiwan

Table S1: Weighting coefficients used in the grid-based single-force (gSF) inversion.

Signal-to-noise ratio (SNR)	Weighted coefficient
SNR ≥ 5.0	1.0
4.5 ≤ SNR < 5.0	0.8
4.0 ≤ SNR < 4.5	0.6
3.0 ≤ SNR < 4.0	0.4
SNR < 3.0	0.1

Table S2: Landquake events detected by the RLMS offline test.

No.	Time of event (UTC)	Long. (°E)	Lat. (°N)	Fitness	F_{max} ($\times 10^{15}$ dyne)	Strike (°)	Dip (°)
1	2009/08/08 06:24	120.60	22.40	0.8300	1.675	47.52	6.52
*2	2009/08/08 09:00	120.77	22.80	0.7711	1.065	78.03	53.29
3	2009/08/08 10:40	120.60	22.60	1.0348	5.217	152.26	30.63
4	2009/08/08 13:12	120.80	22.40	0.6529	2.980	112.53	30.10
*5	2009/08/08 13:55	120.95	23.02	0.6615	1.433	173.94	5.37
6	2009/08/08 17:05	120.80	22.60	0.7672	3.062	115.74	3.10
7	2009/08/08 17:20	121.00	23.20	0.6775	0.798	173.70	30.97
8	2009/08/08 18:44	120.80	22.40	0.9531	2.771	137.22	18.55
9	2009/08/08 19:24	120.60	22.60	0.6037	1.162	158.07	0.99
10	2009/08/08 19:46	120.80	22.40	0.6110	1.275	141.80	6.17
*11	2009/08/08 20:27	120.52	23.13	0.7969	1.918	71.35	27.34
12	2009/08/08 21:29	120.60	23.20	0.7857	2.206	140.06	21.10
13	2009/08/08 21:41	120.80	23.00	0.6887	0.998	178.41	74.81
*14	2009/08/08 22:16	120.70	23.21	0.5968	2.804	249.06	7.15
*15	2009/08/08 23:14	120.78	23.30	0.9897	1.522	57.35	27.39
*16	2009/08/09 00:05	120.99	23.48	0.5929	3.719	359.08	28.25
*17	2009/08/09 00:07	120.95	23.22	0.9804	3.623	92.40	33.00
18	2009/08/09 00:34	120.80	23.20	0.5536	0.671	48.18	17.10
19	2009/08/09 00:48	120.80	23.00	0.7642	0.926	105.67	70.52
*20	2009/08/09 01:26	120.69	22.40	0.6036	1.145	346.48	5.52
21	2009/08/09 01:40	121.20	23.20	0.5590	0.831	109.89	16.01
*22	2009/08/09 02:51	120.76	23.29	0.8752	6.210	104.86	15.99
*23	2009/08/09 03:42	120.88	23.18	0.8445	1.109	241.72	54.81
24	2009/08/09 03:48	120.80	22.40	0.8659	1.136	323.07	13.07
*25	2009/08/09 04:16	120.54	23.15	0.7205	1.238	293.25	18.42
26	2009/08/09 04:24	121.00	23.40	0.6346	0.626	328.50	42.03
27	2009/08/09 05:55	121.00	23.20	0.7623	1.084	97.88	39.88
28	2009/08/09 09:15	120.80	23.00	0.6460	0.792	12.77	23.63
*29	2009/08/09 09:28	120.84	22.32	0.9624	4.056	96.43	42.80
*30	2009/08/09 09:31	120.80	22.56	0.9003	18.570	81.96	30.79
31	2009/08/09 09:33	120.80	22.60	0.8903	6.576	64.46	16.85
*32	2009/08/09 09:52	120.85	22.64	0.6023	0.901	338.22	33.57
33	2009/08/09 10:25	120.80	22.80	0.6470	1.863	114.01	23.25
*34	2009/08/10 03:54	120.74	23.26	0.5509	0.468	14.18	36.50

*35	2009/08/10 11:05	121.04	23.43	0.7784	5.018	249.48	49.04
*36	2009/08/10 11:07	120.94	23.33	1.1114	2.660	74.36	11.10
37	2009/08/10 11:39	120.80	23.40	0.6261	0.358	36.69	29.17
*38	2009/08/10 13:38	120.96	23.15	1.1679	1.955	111.83	13.63
39	2009/08/10 18:02	121.20	23.20	0.6728	4.714	87.78	71.07
*40	2009/08/10 18:42	120.83	23.15	0.9471	0.606	242.01	15.96

* Events with location results obtained from LED

Table S3: Weighting coefficients used in the LED location method.

Normalized cross-correlation coefficient (CC)	Weighted coefficient
$CC \geq 0.85$	1.0
$0.80 \leq CC < 0.85$	0.9
$0.75 \leq CC < 0.80$	0.8
$CC < 0.75$	0.7

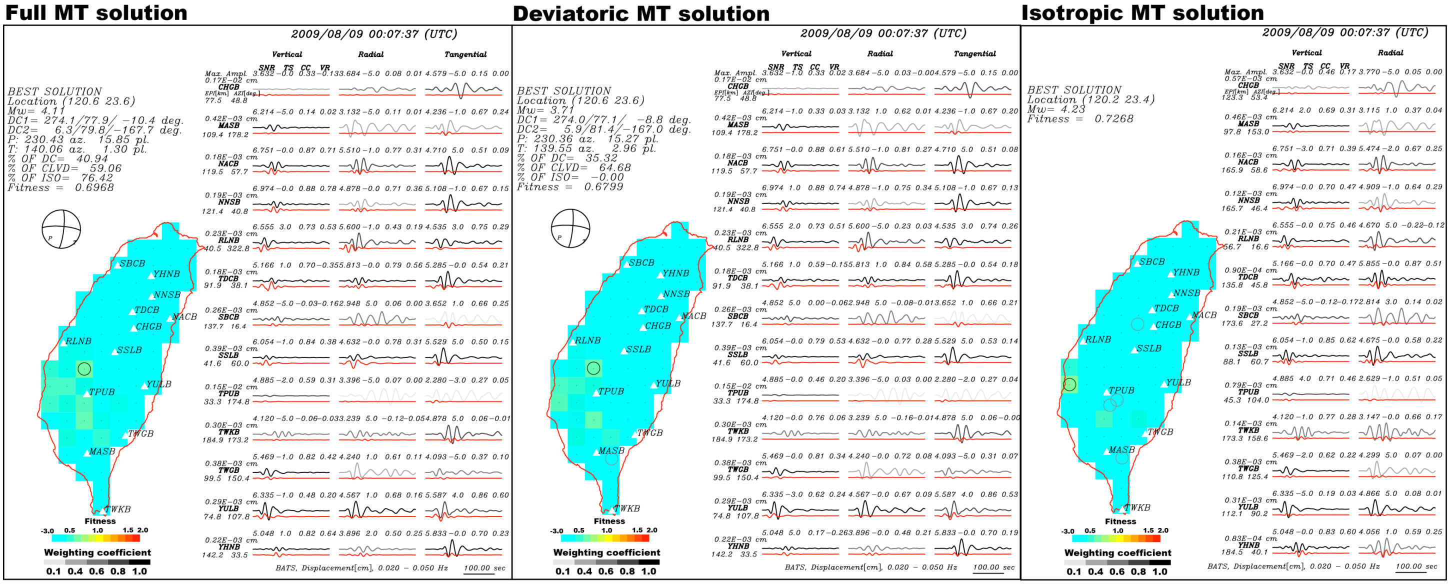

Figure S1: General source inversion (GSI). Examples of fits between records (gray curves) and synthetic seismograms (red curves) calculated for different source mechanisms including full moment tensor (fMT; left panel, fitness: 0.6968), deviatoric MT (dMT, middle panel, 0.6799), and isotropic MT (iMT, right panel, 0.7268). All waveforms are bandpass filtered to 0.02-0.05 Hz. Maps are created using GMT (Generic Mapping Tools, http://gmt.soest.hawaii.edu/; Supplementary ref. 1) software.

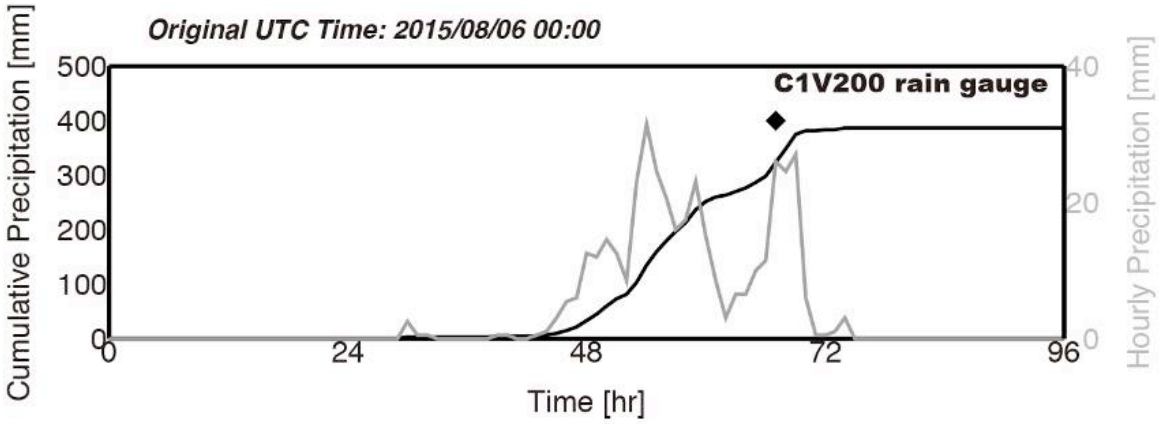

Figure S2: Rainfall data at rain gauge station C1V200. Grey and black traces show time series for the precipitation rate and cumulative rainfall, respectively. Diamond represents a detected landquake event.

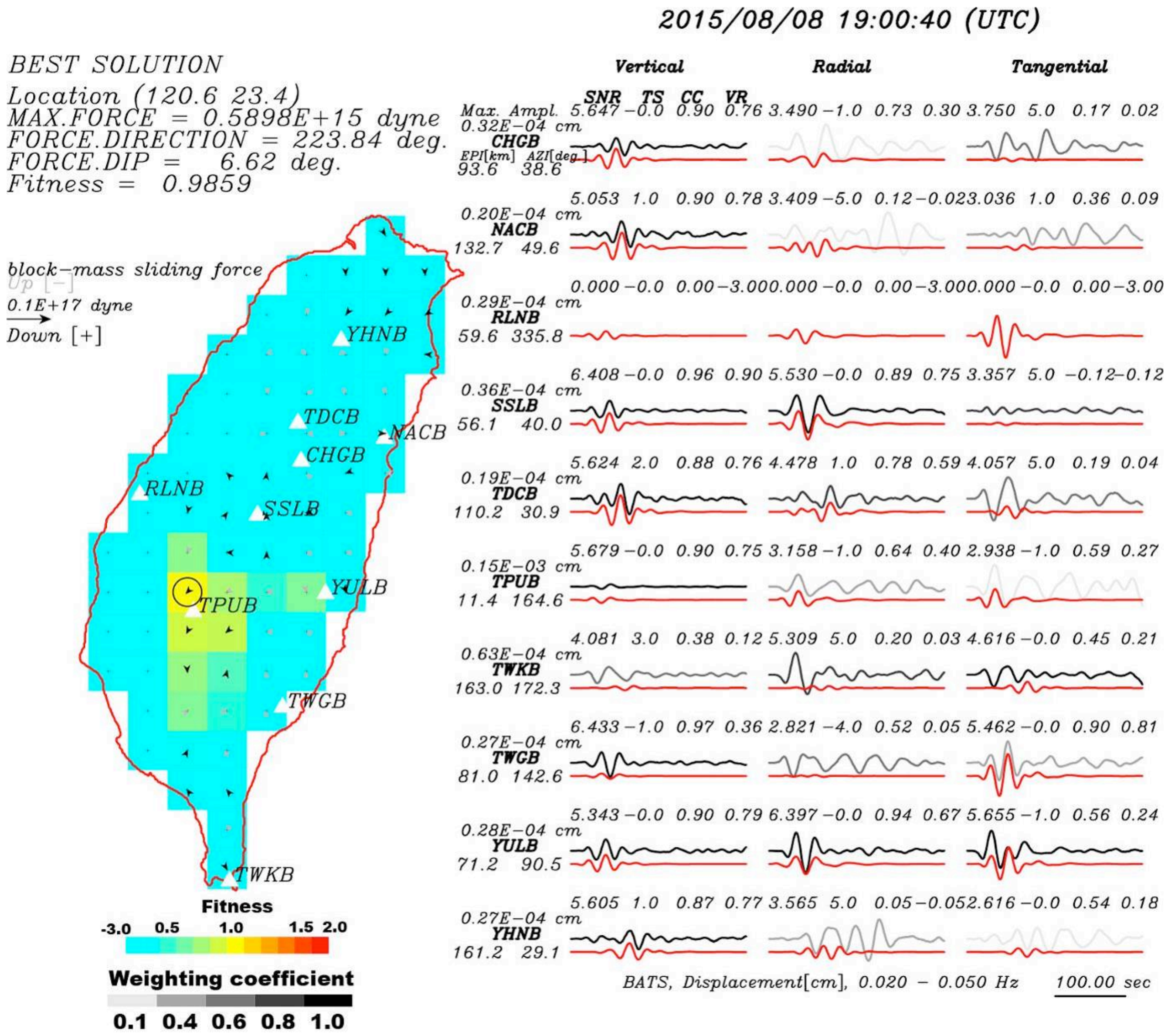

Figure S3: An online real-time gSF inversion result. Grid-based single force (gSF) inversion for an event automatically detected by the online RLMS during the 2015 Typhoon Soudelor passage. The left panel summarizes results of source location, maximum force magnitude (F_{max}), direction and dip of the sliding force. Black circle depicts the location with the largest fitness value of 0.9859. The color scale shows the fitness value. Right panel shows the waveform fits between observed and synthetic seismograms calculated for the best solution of SF mechanism. Different grey levels indicate different weighting factors based on the signal-to-noise ratios (SNR) of observed traces for each component. All waveforms are bandpass filtered to 0.02-0.05 Hz. The maximum amplitude, station name, epicentral distance and station azimuth are given at the start of each row. The SNR value, time shift (TS), and the normalized cross-correlation coefficient (CC) and variance reduction (VR) are given at the top of each trace. Map is created using GMT (Generic Mapping Tools, http://gmt.soest.hawaii.edu/; Supplementary ref. 1) software.

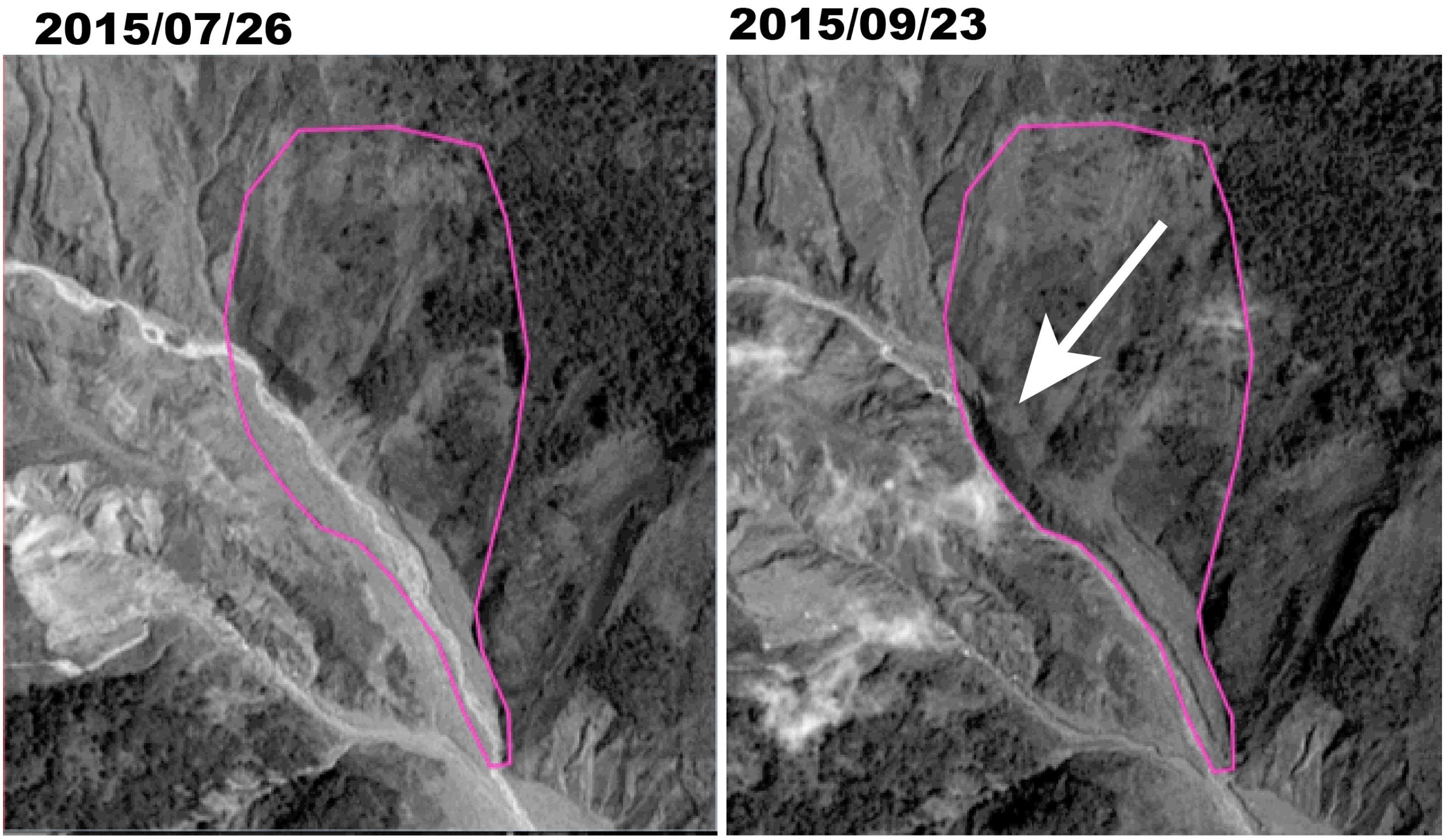

Figure S4: FORMOSAT-2 images derived by the Center for Space and Remote Sensing Research (CSRSR; http://www1.csrsr.ncu.edu.tw/Ver13_J30/), with a spatial resolution of 2 m before and after Typhoon Soudelor. Arrow depicts the sliding force direction of Soudelor landquake event determined in this study.

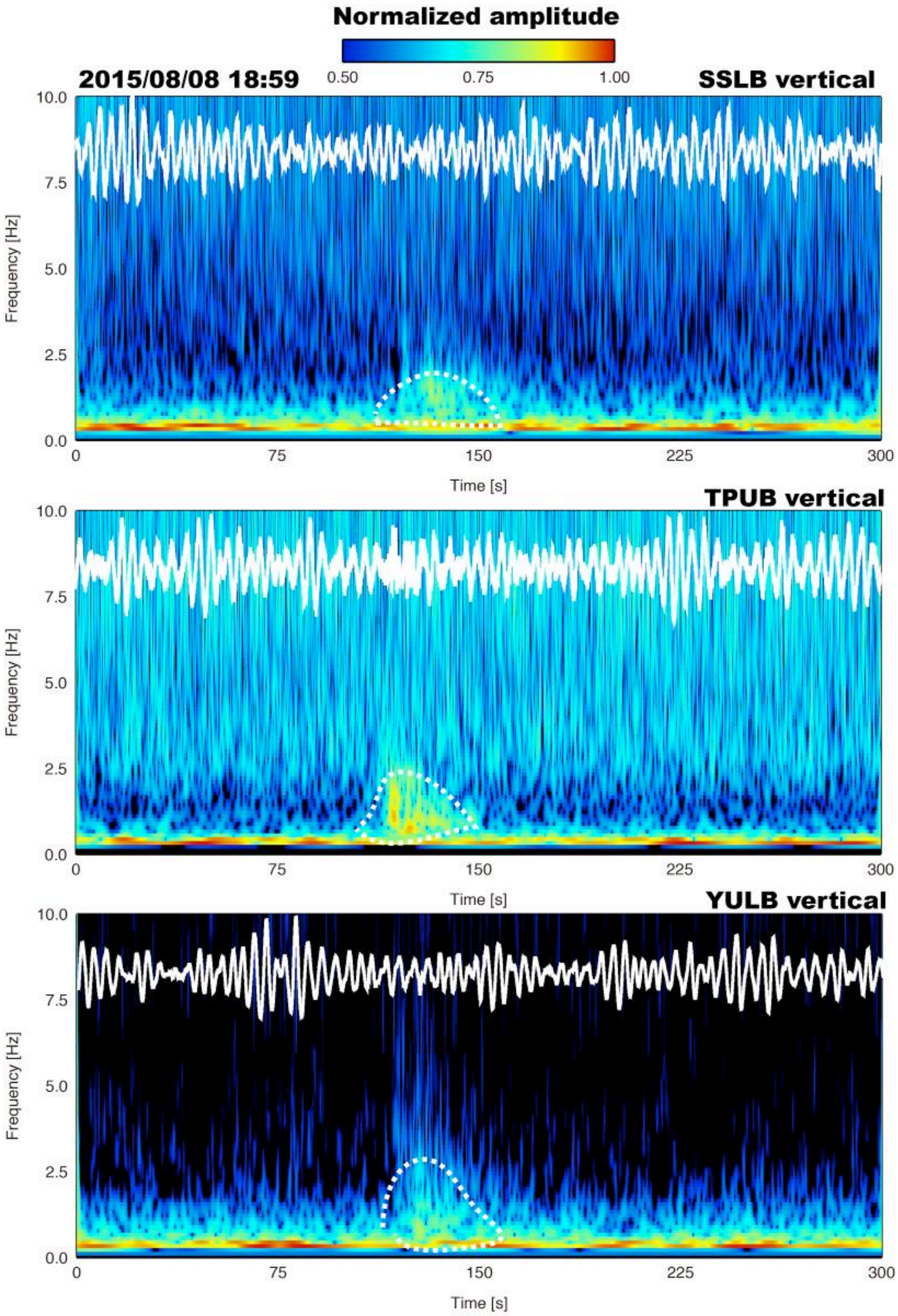

Figure S5: Spectrograms for an event detected during Typhoon Soudelor passage. White traces are the original vertical-component velocity seismograms. On the time-frequency plane the highest concentrations of energy arriving from the landquake event are indicated by the white dashed line. The color scale is such that the maximum normalized amplitude is depicted in red while black indicates normalized amplitudes less than 0.5.

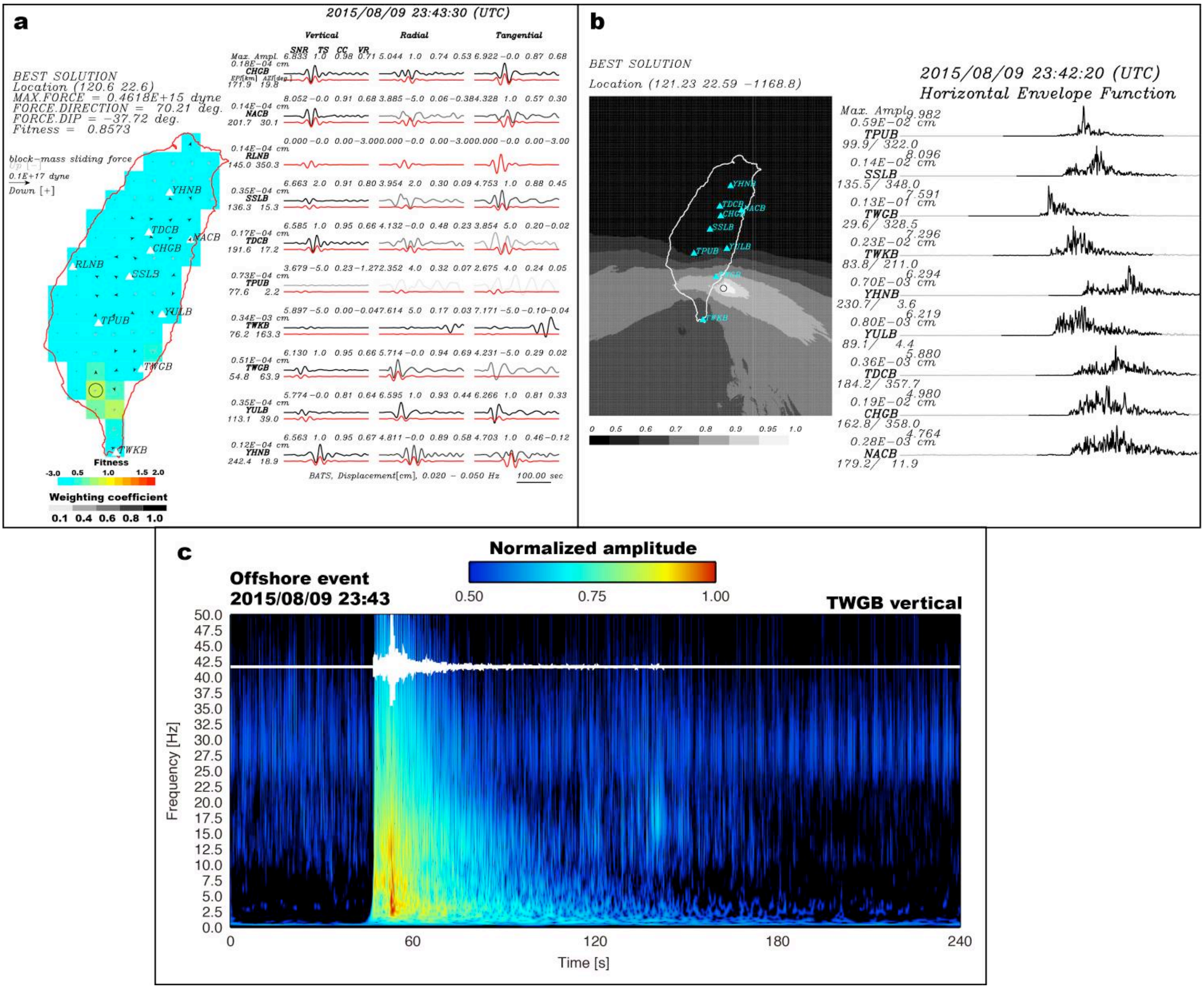

Figure S6: Results of gSF and LED for an offshore earthquake event. SF mechanism and location are determined from (a) gSF inversion and (b) LED, respectively. The RLMS incorrectly clarifies an offshore earthquake event as inland landquake by using GSI procedure. (c) Spectrograms at BATS station TWGB for an offshore event. White trace is the original vertical-component velocity seismogram. The color scale is such that the maximum normalized amplitude is depicted in red while black indicates normalized amplitudes less than 0.5. Maps are created using GMT (Generic Mapping Tools, http://gmt.soest.hawaii.edu/; Supplementary ref. 1) software.

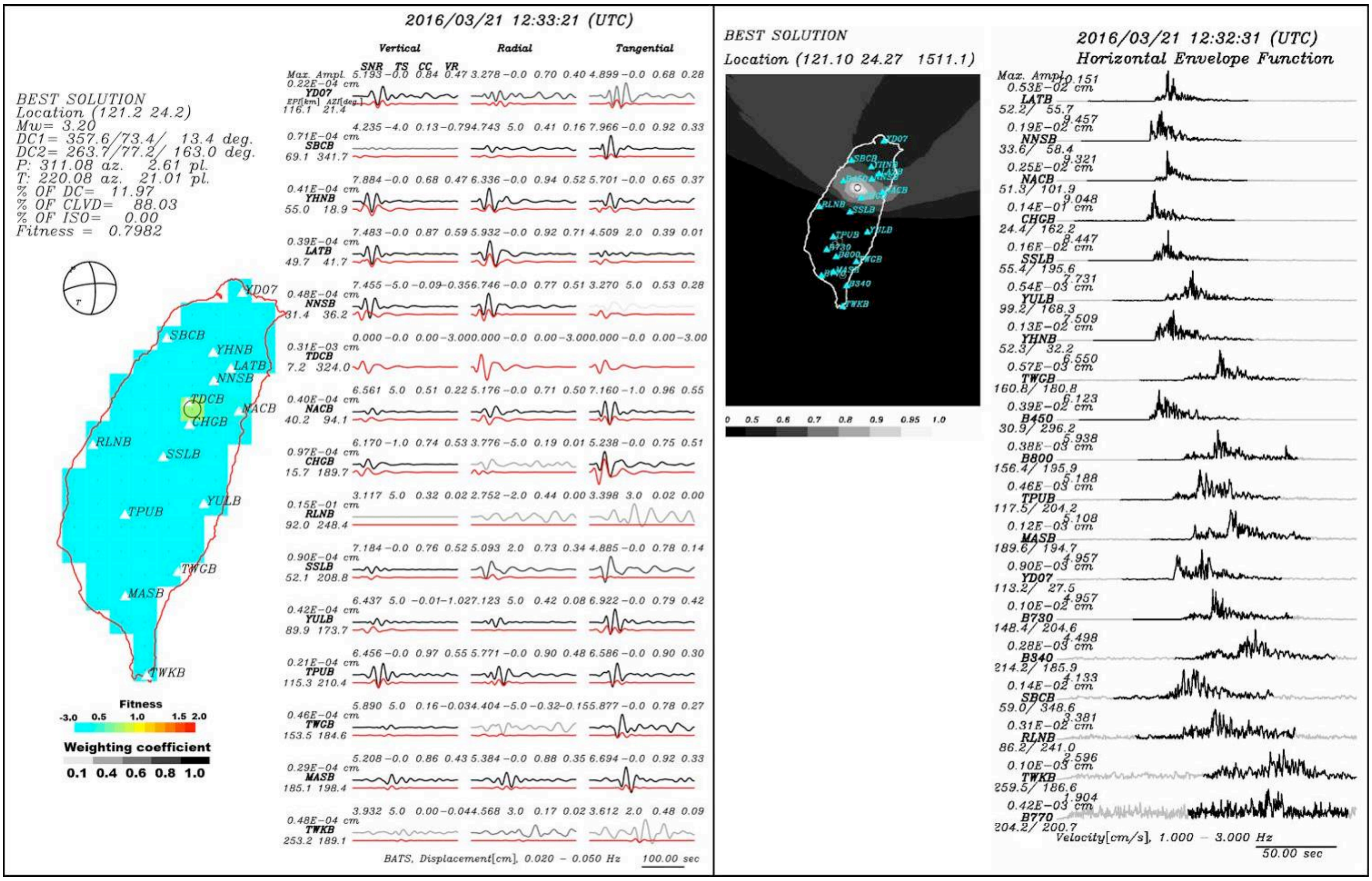

Figure S7: LED result for an earthquake event. Focal mechanism and location are determined from general source inversion (GSI) of waveforms (left) and LED (right), respectively. Our RLMS identifies this event as an earthquake, consistent with an event reported by the real-time moment tensor (RMT) determination system (http://rmt.earth.sinica.edu.tw/earthquake/eq_20160321123321.png, last accessed May 2016), which routinely monitors earthquakes in Taiwan. Maps are created using GMT (Generic Mapping Tools, http://gmt.soest.hawaii.edu/; Supplementary ref. 1) software.

Supplementary references

1. Wessel, P. et al. Generic Mapping Tools: Improved Version Released. *EOS Trans. AGU* **94(45)**, 409-410 (2013).
